# Analysis of genetic recombination and the pan-genome of a highly recombinogenic bacteriophage species

**DOI:** 10.1099/mgen.0.000282

**Published:** 2019-07-16

**Authors:** Koji Yahara, Philippe Lehours, Filipa F. Vale

**Affiliations:** ^1^ Antimicrobial Resistance Research Center, National Institute of Infectious Diseases, 4-2-1 Aobacho, Higashimurayama, Tokyo 189-0002, Japan; ^2^ French National Reference Center for Campylobacters and Helicobacters, Bordeaux, France; ^3^ University of Bordeaux, INSERM, UMR1053 Bordeaux Research in Translational Oncology, BaRITOn, 33076 Bordeaux, France; ^4^ Host–Pathogen Interactions Unit, Research Institute for Medicines (iMed-ULisboa), Faculdade de Farmácia da Universidade de Lisboa, Lisboa, Portugal

**Keywords:** homologous recombination, bacteriophage, pan-genome, core genome, *Helicobacter pylori*, evolution

## Abstract

Bacteriophages are the most prevalent biological entities impacting on the ecosystem and are characterized by their extensive diversity. However, there are two aspects of phages that have remained largely unexplored: genetic flux by recombination between phage populations and characterization of specific phages in terms of the pan-genome. Here, we examined the recombination and pan-genome in *Helicobacter pylori* prophages at both the genome and gene level. In the genome-level analysis, we applied, for the first time, chromosome painting and fineSTRUCTURE algorithms to a phage species, and showed novel trends in inter-population genetic flux. Notably, hpEastAsia is a phage population that imported a higher proportion of DNA fragments from other phages, whereas the hpSWEurope phages showed weaker signatures of inter-population recombination, suggesting genetic isolation. The gene-level analysis showed that, after parameter tuning of the prokaryote pan-genome analysis program, *H. pylori* phages have a pan-genome consisting of 75 genes and a soft-core genome of 10 genes, which includes genes involved in the lytic and lysogenic life cycles. Quantitative analysis of recombination events of the soft-core genes showed no substantial variation in the intensity of recombination across the genes, but rather equally frequent recombination among housekeeping genes that were previously reported to be less prone to recombination. The signature of frequent recombination appears to reflect the host–phage evolutionary arms race, either by contributing to escape from bacterial immunity or by protecting the host by producing defective phages.

## Data Summary

1. The genome accession numbers and metadata, including phage population and information from multilocus sequence typing (MLST), are in Table 1.
2. The protein ID for the phage pan-genome is in Table S2 (available in the online version of this article).

**Impact Statement**

Despite the large number of bacteriophages (phages) present in the biosphere, little is known about genetic flux by recombination between populations of phages, and the variation in intensity of recombination across phage genes in general. We studied these aspects using genome sequences of phages infecting a highly recombinogenic bacterial species, *Helicobacter pylori *. Our genome-level analysis showed for the first time the extent and direction of the genetic flux among phage populations, including those that show significantly stronger or weaker signatures of genetic flux. In the subsequent gene-level analysis, we found that parameter tuning of the standard software was necessary to identify well-conserved genes, and it showed equally high recombination among them, including genes that were reported to be less prone to recombination. Our findings suggest that *H. pylori* phages are among the most recombinogenic phages, reflecting coevolutionary relationships with their hosts.

**Table 1.** Phage genomes used in the recombination analysis

Genome	Phage population*	Bacterial population†	Genome length (bp)	Accession no.	References
Hac	na	na	28 420	AM260522.1	[1]
KHP30	hpEastAsia	na	26 215	NC_019928.1	[2]
KHP40	hpEastAsia	na	26 449	NC_019931.1	[2]
1961P	hpEastAsia	na	26 836	NC_019512.1	[3]
Fr-B58-M	hpEastAsia	hpEastAsia	22 559	SRP071277 KX119193	[4]
Pt-B92-G	hpAfrica1	hpEurope	30 548	SRP071282 KX119197	[4]
Pt-212–99 R-U	hpAfrica1	hpEurope	23 008	SRP071292 KX119189	[4]
Pt-1293-U	hpAfrica1	hpEurope	30 071	SRP071280 KX119202	[4]
Pt-5771-G	hpAfrica1	hpEurope	29 801	SRP064707 KX119199	[4]
Pt-B89-G	hpAfrica1	hpEurope	27 363	SRP071278 KX119203	[4]
Pt-5322-G	hpAfrica1	hpEurope	28 341	SRP071284 KX119198	[4]
Fr-ANT170-U	hpAfrica1	hpEurope	31 200	SRP072438 KX119201	[4]
Fr-MEG235-U	hpAfrica1	hpEurope	31 236	SRP072439 KX119200	[4]
Pt-1846-U	hpAfrica1	hpEurope	27 960	SRP071062 KX119176	[4]
Pt-228_99 G	hpAfrica1	hpEurope	30 078	SRP071067 KX119175	[4]
phiHP33	hpAfrica1	hpEurope	24 645	AFAO00000000.1 NC_016568.1	[5]
Pt-4481-G	hpAfrica1	hpEurope	25 388	SRP071279 KX119196	[4]
UK-EN31-U	hpNEurope	hspEuropeN†	30 456	SRP071274 KX119174	[4]
UK-EN32-U	hpNEurope	hspEuropeN†	29 882	SRP071276 KX119206	[4]
De-M53-M	hpNEurope	hspEuropeN†	28 068	SRP064710 KX119205	[4]
India7	hpNEurope	hpAsia2†	28 310	CP002331.1	
Cuz20	hpNEurope	hspAmerind†	28 587	CP002076.1	
Sw-A626-G	hpEuropeN	hspEuropeN†	30 977	SRP071294 KX119177	[4]
Sw-577-G	hpNEurope	hspEuropeN†	26 906	SRP071293 KX119204	[4]
Fr-G12-G	hpSWEurope†	hpEurope	28 565	SRP064708 KX119194	[4]
Pt-4472-G	hpSWEurope	hspEuropeS†	27 572	SRP071271 KX119190	[4]
Fr-GC43-A	hpSWEurope†	hpEurope	32 975	SRP072440 KX119195	[4]
Pt-1918-U	hpSWEurope	hspEuropeS†	28 670	SRP064706 KX119192	[4]
Pt-4497-U	hpSWEurope	hspEuropeS†	29 393	SRP064709 KX119192	[4]
Fr-B41-M	hpSWEurope	hspEuropeS*	29 388	SRP072441 KX119188	[4]

*The phage population was determined by phage sequence typing (PST) using two phage genes [6].

†The bacterial population was determined using seven MLST genes [6]. The asterisks indicate subpopulations determined by fineSTRUCTURE (Fig. S4) [7]. na, not applicable, phage genome only or non-pylori *Helicobacter* phage.

## Introduction

Viruses are the most prevalent biological entities on Earth; they play important roles in ecological balance and are characterized by their extensive diversity [8]. Interactions between bacteria and bacterial viruses, also known as bacteriophages or phages, help to shape the population structure of microbial communities, as well as their ecology and evolution [9]. Phages acquire and donate genes from other phage or bacterial genomes, and thus play an important role in the evolution, physiology and pathogenicity of their bacterial hosts [9]. The high genetic diversity in phages is likely due to their ancient origin (they originated about 3 billion years ago), the huge number of phages present in the biosphere and their frequent infection of permissive hosts. These lead phages to encounter DNA derived from bacteria or prophages (phages integrated into bacterial chromosomes) with which they can recombine [10]. Such recombination is a driving force of evolution, which can occur either through homologous recombination or non-homologous site-specific recombination, generating a mixture of recombinant types. However, as a consequence of such recombination, most recombinants are defective for growth, and natural selection eliminates all but a very small minority in which biological functions are intact, giving rise to a population with non-random recombination sites [10].

*Helicobacter pylori* is notable among the bacterial host species of phages because it is a long-term colonizer of the human stomach, it has been co-evolving with its human host for more than 100 000 years [11] and it is highly recombinogenic [12, 13]. About 20 % of *H. pylori* strains contain prophage genes [4, 6, 14, 15], which are present in a phylogeographical pattern that corresponds to that of the host bacterium [6, 14] and provide additional insights into the population structure. For example, the hpEurope population has two distinct subgroups, northern and southwestern, that are segregated by only two prophage genes [6]. Likewise, a comparison of *H. pylori* whole genomes also pointed to the existence of two European subpopulations [7]. *H. pylori* phages are highly diverse. They have conserved synteny marked by punctuated gene loss, the introduction of insertion sequences that do not perturb gene synteny and diverse insertion sites with some common traits among *H. pylori* populations [4]. Mobile elements such as phages may contribute to *H. pylori* pathogenicity [16–18], which has been suggested by the correlation of prophage-related sequences with the presence of the virulence genes *cagA* and *vacA* [19].

Previous studies of recombination events in *H. pylori* led to a better understanding of genetic flux and showed that subgroups of the Amerind and East Asian origin populations import a significantly smaller number of fragments than they export to other populations [20], and that *H. pylori* strains from the American continent originated from an admixture between *H. pylori* strains of European and African origin, without extensive input from pre-Columbian (hspAmerind) strains [7]. In contrast, little is known about the genetic flux between populations of phages in general. In addition, although a recent study showed evidence of recombination or genome mosaicism within some *H. pylori* prophages [4], little is known about the variation in intensity of recombination across phage genes, as was recently shown in various phages through an analysis of the Earth’s virome [21].

Another aspect of phage biology that is in general poorly understood is the question of how specific phages can be characterized in terms of their pan-genome. The pan-genome corresponds to the entire set of genes in the genomes within a species, while the core genome is the set of genes that are present in all genomes [22]. Notably, phages do not have such a core genome that is conserved among all phages across their phylogeny [23]. The diversity in phage genomes, notable by the presence of genes in many phages with low sequence similarity [24], makes it difficult to study phage core genomes. To overcome this limitation, we conducted analyses based on both genome alignment at the synteny level and gene-by-gene alignment at a finer level. Regarding the latter, we propose parameter tuning of the standard prokaryote pan-genome analysis program, and using the soft-core genome, which is defined here as the genes present in >90 % of the genomes, to verify sequence similarity. We applied this approach to *H. pylori* prophages, which have preserved genome synteny and a probable modular constitution [4], as little is known about their pan-genome and their genetic diversity.

To explore these two aspects, we conducted a study of recombination in *H. pylori* prophages at both the genome and gene (soft core) levels using 29 *H *. *pylori* genomes and 1 *Helicobacter acinonychis* genome carrying prophages >20 kb. The genome-level approach was used to elucidate the genetic flux of imported and exported sequences in distinct populations of prophages and their host bacteria. The soft-core gene-level approach was used to examine whether some genes show signatures of increased recombination based on a determination of the soft-core genome and pan-genome.

## Methods

### Genome sequences, alignment and prediction of prophage activity

We prepared 1 *H. acinonychis* and 29 *Hh*. *pylori* complete prophage sequences (>20 kb) (Table 1), which (i) are present as a single fragment based on Sanger sequencing closing of the genome [1, 3–5, 25] and (ii) have previously been assigned to a population by prophage sequence typing (PST) [6]. The complete prophage genomes were aligned at the synteny level with MAFFT (version 7) [26]. We excluded identical genomes (i.e. UK-EN32-U, which is identical to UK-EN-31-U, and Fr-MEG235-U, which is identical to Fr-ANT170-U) and a genome with a large inversion (Pt-4481-G), resulting in 27 (=29+1–3) complete prophage genome sequences. We excluded the genome with a large inversion because the inverted region did not align well, and the chromosome painting algorithm does not allow missing data in the alignment. The Prophage Hunter tool [27] was used to predict prophage activity, i.e. whether the prophage could be induced into a lytic cycle.

### Analysis of the population structure and genetic flux in *H. pylori* phage genomes

We inferred the population structure of *H. pylori* phages and the genetic flux between populations from phage genome haplotype data (i.e. SNPs without missing data and their positions in the genome alignment) using chromosome painting and fineSTRUCTURE algorithms, according to a procedure used in our previous study of *H. pylori* genomes [7, 20]. The chromosome painting algorithm is a hidden Markov model (HMM) to ‘paint’ a ‘recipient’ haplotype as a series of recombination-derived DNA fragments (‘chunks’) from a panel of ‘donor’ haplotypes from other individuals in the sample based on sequence similarity between donor and recipient. The interpretation of the painting is that the donor at a given region of the genome has the most recent shared common ancestor with the recipient individual amongst all of the possible donors in the panel. Changes in the identity of the donor along the sequence reflect recombination events that led to different genealogical histories for different parts of the genome [28, 29] . Lawson *et al*. used the algorithm to summarize information for genome-wide SNPs into chunks based on a ‘co-ancestry matrix’ that tabulated the number of chunks from each donor to each recipient individual. The data reduction from a haplotype matrix to a co-ancestry matrix enables model-based clustering using ﬁneSTRUCTURE. The two-step approach was demonstrated to be effective not only in humans, but also in *H. pylori *. We used ChromoPainter (version 0.04) to run the chromosome painting algorithm separately for different recipient individuals. Then, fineSTRUCTURE (version 0.02) was used to run 100 000 iterations of both the burn-in and Markov chain Monte Carlo (MCMC) chain to cluster individuals based on the co-ancestry matrix.

### Prophage core- and pan-genomes

The core- and pan-genomes of *H. pylori* prophages were determined using Roary [30]. The *H. acinonychis* prophage was not included in the pan-genome analysis because *H. acinonychis* is a non-pylori *Helicobacter *. Because of the extreme diversity typically found in phages, −*i* (the minimum percentage identity of the shorter length for blastp) was set at 90, 80, 70, 60, 50 or 40 with or without the −*s* option, for splitting or not splitting paralogues, respectively. This approach was used to find the most suitable parameters for determining the phage pan-genome by overcoming the limitations of high diversity; the existence of stop codons followed by a second start codon, leading to the annotation of two genes or a smaller gene, which hinders determination of the percentage identity and leads to the misidentification of similar genes; or other annotation limitations, such as the non-identification of overlapping genes, the misidentification of unique genes in a particular genome, or genes that are highly divergent from known homologues [31].

### Analysis of recombination at the gene level

**Fig. 1.**
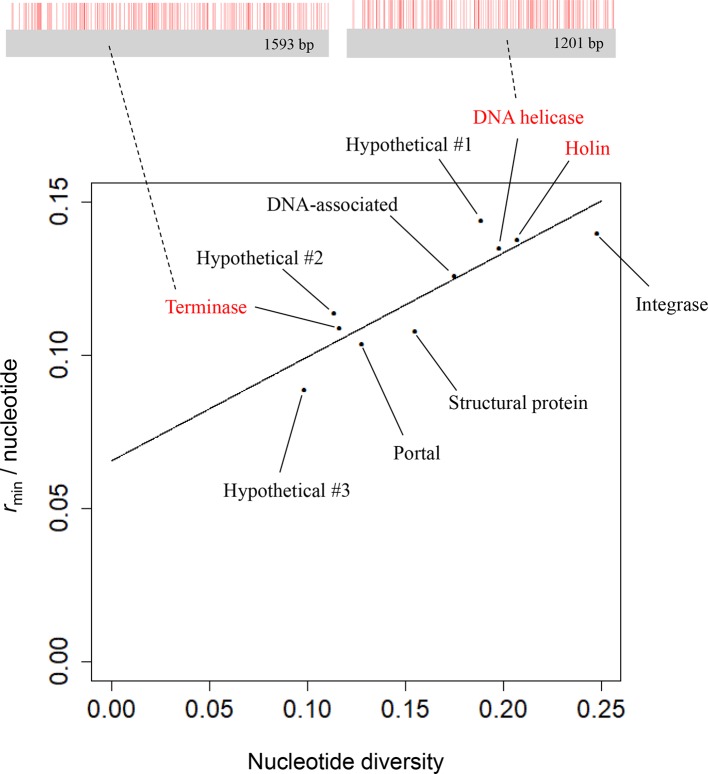
Relationship between the minimum number of recombination events (*r*_min_) per gene length and nucleotide diversity among the *H. pylori* phage soft-core genes. The *x* and *y* axes are nucleotide diversity and *r*_min_ per nucleotide, respectively. The solid line indicates the linear regression between nucleotide diversity and the minimum number of recombination events. Recombination breakpoints in the genes encoding DNA helicase and terminase are shown as red vertical bars at the top.

Recombination analysis was carried out at the gene level to determine the genes in the soft-core genome (genes present in at least 90 % of the genomes), which were identified using the Roary pipeline [30] with the −*i* 70 and −*s* options. The genes belonging to the soft-core genome that were analysed are integrase, portal protein, structural protein, DNA helicase, terminase, holin, hypothetical protein/DNA-associated and three other hypothetical proteins. For each gene, the minimum number of recombination events (*r*_min_) was calculated using the four-gamete test [32], which locates pairs of the closest segregating sites within four haplotypes that are likely to be generated by recombination. We used the method implemented in the PGEToolbox [33], which filters gaps in advance. Basic population genetic statistics (e.g. nucleotide diversity) were also calculated for each gene using DnaSP v5 [34]. Genes with stop codons in the middle of the sequence were excluded from this analysis. We then conducted multiple linear regressions to capture the overall relationship between the *r*_min_ per nucleotide and nucleotide diversity after controlling for differences in the number of individuals: *y*_*i*_ *=* *β*_*0*_ *+* *β*_1_ *x*_1_ *_,_*_*i*_ *+* *β*_2_ *x*_2_ *_,_*_*i*_ *+* *ε*_*i*_, where for gene *i*, *y_i_* is the minimum number of recombination events per nucleotide; *x_1,i_* is nucleotide diversity; *x_2,i_* is the number of aligned sequences; *β*_0_ is the intercept; *β*_1_ and *β*_2_ are regression coefficients; and *ε*_*i*_ is error, which is normally distributed. We plotted the regression line in Fig. 1 given the parameter estimates, holding constant *x_2_* as the average number of aligned sequences in a gene.

## Results

### Recombination and population structure of phage genomes

**Fig. 2.**
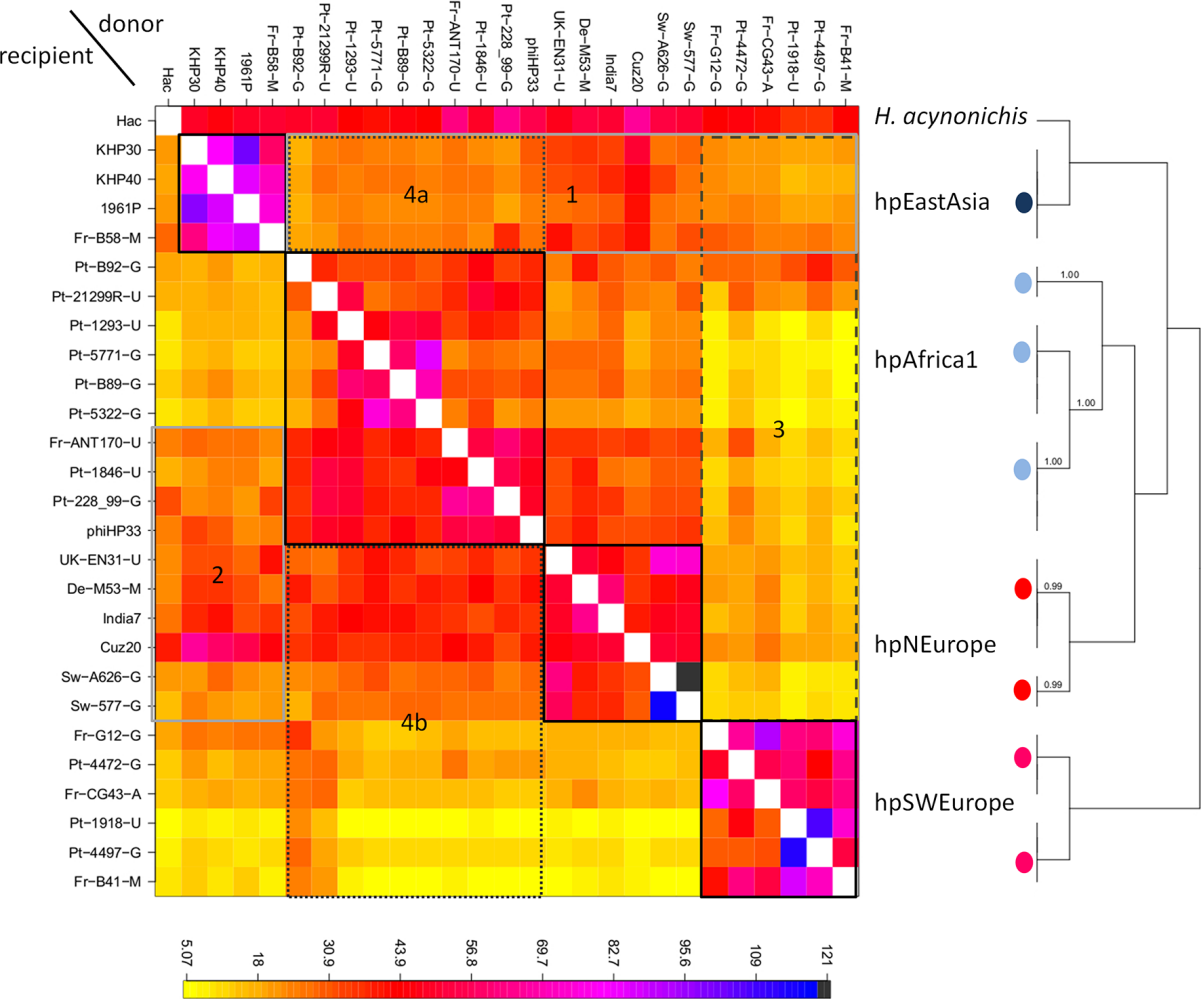
Co-ancestry matrix of *H. pylori* phage genomes. Each lane represents the genome of a phage shown on the left. The colour of each square of the matrix represents the expected number of fragments exported from a donor genome (column) to a recipient genome (row). These genomes belong to the phage populations shown on the right. The tree obtained from the fineSTRUCTURE analysis shows the subgroups of each population. The subgroups of each phage population are indicated by coloured circles as follows: hpEastAsia, dark blue; hpAfrica1, light blue; hpNEurope, red ; and hpSWEurope, pink (recipient prefixes: 'De', German; 'Fr', France; 'Pt', Portugal; 'Sw', Sweden; 'UK', United Kingdom).

For 27 *H *. *pylori* prophages, 19 of which were predicted to be active using Phage Hunter [27] (Table S1), each complete genome was reconstructed using fragments (‘chunks’) of DNA donated by other individual genomes using the chromosome painting algorithm, as summarized and visualized in the co-ancestry matrix (Fig. 2). The algorithm was applied to 7618 core SNPs and their positions in the alignment (53266 bp) of the prophage genome sequences. Based on the matrix, individual phages were assigned to subgroups by the fineSTRUCTURE clustering algorithm. The two-step approach is applicable even when the sample size is small, as shown previously in *H. pylori *, which is in contrast to STRUCTURE, which is based on allele frequency and thus requires at least 15–20 individuals per hypothesized population [20]. This approach revealed a finer population structure for the genomes classified as hpAfrica1, presenting three subgroups (light blue circles in Fig. 2), and for the genomes in the hpNEurope and hpSWEurope populations (designated in our previous study [6]), each presenting two subgroups (red and pink circles in Fig. 2, respectively). It also revealed notable signatures of inter-population recombination or gene flow, namely, that the hpEastAsia genomes are recipients of DNA chunks from hpAfrica1, hpNEurope and hpSWEurope (box 1 in Fig. 2), while two of the hpAfrica1 subgroups (top and middle light blue circles in Fig. 2) are recipients of DNA chunks from genomes of hpNEurope origin, and the other subgroup received DNA fragments from both hpNEurope and hpEastAsia genomes. In addition, this method revealed that hpNEurope genomes are recipients of DNA from genomes of African and East Asian origin, which is clearer in the genomes of phages from northern continental Europe (top red circle in Fig. 2) than in the genomes of Scandinavian origin (bottom red circle in Fig. 2) (see the Discussion for more quantitative examination and interpretation). Such signatures of inter-population recombination or gene flow were weaker in hpSWEurope genomes, which might be regarded as an outgroup in the *H. pylori* phages. The *H. acinonychis* prophage genome, Hac, also seems to show such signatures; however, this is an artifact seen in a sequence that is much more divergent than the others.

### Classifying the *H. pylori* prophage soft core- and pan-genome genes

For the pan-genome analysis of the prophage genomes, given the high diversity, we tested several combinations of parameters related to the percentage sequence identity (sequence identity from 40 to 90 %), with and without paralogue disabling (Fig. S1). The default parameters for the prokaryote pan-genome analysis program, i.e. 90 % percentage identity and paralogue enabled, yielded no core genes, which contradicted previous results, since it was previously known that these genomes shared genes (see below). Indeed, at sequence percentage identity values greater than 70, the size of the pan-genome increased exponentially. While no genes were classified as core or soft core, genes such as integrase were present in every single prophage genome (see Fig. S2, which illustrates the genome alignment, location and alignment of the integrase gene). A closer inspection of the results with or without paralogue split, revealed that genes considered to be paralogues were in fact genes with a stop codon and an initiation codon in the middle of the gene, and they did not correspond to true gene duplications. Using a minimum sequence identity of 70 % with paralogue split disabled, the pan-genome has 75 genes; 13 % (10/75) of these genes belong to the soft-core genome, and 21 % (16/75) of the genes are singletons present in a single genome. A DNA primase gene was present in all phage genomes, but in two different clusters of orthologous genes, and thus was not considered to be a soft-core gene. Most (75 %, 12/16) singletons had unassigned functions, while 30 % of the soft-core genes had no functional assignment (Table S2).

### Recombination among phage soft-core genes

For each soft-core gene, we calculated the minimum number of recombination events (*r*_min_) using the four-gamete test, a conservative method for locating pairs of the closest segregating sites within four haplotypes that were likely to have been generated by recombination. The *r*_min_ per nucleotide was at least 0.089, which was higher than the highest value (0.053) among the 211 viral (phage species) groups using the Earth’s virome data [21]. Although the value itself depends on nucleotide diversity, it suggests frequent recombination among the *H. pylori* phages, which is similar to the host bacterium. To account for the dependence of the *r*_min_ per nucleotide on nucleotide diversity, we took an approach similar to that used in previous studies [21, 35]. We plotted the *r*_min_ per nucleotide versus the nucleotide diversity of a gene and calculated the linear regression that captured the overall relationship between nucleotide diversity and the minimum number of recombination events per nucleotide (Fig. 1). The extent of deviation from the regression line is a measure of the intensity of recombination after accounting for nucleotide diversity. Overall, the soft-core genes did not show a clear deviation from the regression line, suggesting the absence of substantial variation in the intensity of recombination across phage genes. In contrast, housekeeping genes encoding holin, DNA helicase and terminase (red in Fig. 1), which were reported to be less prone to recombination [36, 37], were similarly close to the regression line, suggesting equally frequent recombination among genes.

## Discussion

This is the first study to apply chromosome painting and fineSTRUCTURE to prophage genomes. This approach allowed us to elucidate the extent and direction of the genetic flux among distinct phage populations. The phage populations we identified were consistent with those determined by phage typing using integrase and holin genes [6]. However, we uniquely identified subgroups within these populations and genetic flux among these subgroups (Figs 1 and S3).

Notably, the hpEastAsia phage population imported nearly the same percentages of DNA fragments from all other populations (median: 24.4 % per individual recipient), which differed from the percentages of imports that other populations received from different populations (median: 13.2 %, *P* value=0.017, Wilcoxon’s rank sum test; box 1 in Fig. 2). The hpEastAsia phage population exported more DNA fragments to the hpNEurope population and one of the hpAfrica1 subgroups (median exports of hpEastAsia phages to hpNEurope and subgroup of hpAfrica1 : 12.8 %; box 2 in Fig. 2) than to hpSWEurope and the other subgroups of hpAfrica1 (median: 10.0 % per donor population; *P* value=1.3e-11, Wilcoxon’s rank sum test). This pattern was observed among phages in the hpEastAsia population isolated in both East Asian countries (KHP30, KHP40 and P1961) and Europe (Fr-B58-M). This result seems to contradict the findings of a previous report showing that hpEastAsia *H. pylori* genomes imported a smaller number of fragments from other populations than they exported [20]. One hypothesis is that hpEastAsia phages have been exposed to more phages from other populations than the phages in other populations have been exposed to hpEastAsia phages.

The hpSWEurope phage population exported fewer DNA fragments to other phage populations (median exports of hpSWEurope to other populations: 11.9 %; box 3 in Fig. 2) than other populations did (median: 21.3 % per donor population; *P* value<2.2e-16, Wilcoxon’s rank sum test). The two observable groups of hpSWEurope phages show signs of genetic isolation, which was previously suggested by the long branches in the phylogenetic trees [4], especially for the subgroup including phages Fr-G12-G, Pt-4472-G, Fr-GC43-A, Pt-1918-U, Pt-4497-G and Fr-B41-M, in which the host belongs to the hpEurope population.

There is a bidirectional genetic flux between the hpNEurope and hpAfrica1 phage populations; however, it should be noted that the hpAfrica1 strains were isolated in Europe, and thus may be more exposed to other European phages, which could influence the results. Although isolated in Europe, these strains were originally defined as hpAfrica1 based on the typing of the integrase and holin genes, and also because they were previously shown to cluster with other *H. pylori* hpAfrica1 prophages isolated from African countries [6, 14]. It has been suggested that directional gene flow may indicate that a group of genomes act as donors during recombination events, representing a reservoir for diversity [38], a role that appears to be played by hpAfrica1 phages, acting as substantial donors to other populations. Indeed, hpAfrica1 phages exported significantly more DNA fragments (median exports of hpAfrica1 to other populations: 30.2 %; boxes 4a and 4b in Fig. 2) than other populations did (median: 12.6 % per donor population; *P* value=0.0001, Wilcoxon’s rank sum test).

Chromosome painting and fineSTRUCTURE algorithms were applied to the 7618 core SNPs and their positions in the alignment of the prophage genome sequences. If the number of SNPs were too small, the co-ancestry matrix (heatmap) would become uniform because each recipient genome would have similar, and small, numbers of fragments (chunks) of DNA inferred to be donated by other donor individuals. Clearly, that is not the case, as observed in the co-ancestry matrix in Fig. 2, which shows variation in the number of fragments (chunks) of DNA for each recipient genome and population structure. Therefore, even though the prophage genomes are much smaller than those of the host, the current dataset has sufficient information for the algorithms to work.

Although no genomic study has yet quantified recombination, phages have generally been considered as actively engaged with horizontal genetic exchange (recombination between different individuals), and their genomes are pervasively mosaic in their architectures [39]. In this context, our study contributes in general to phage biology by (1) showing the extent and direction of the genetic flux among phage populations based on the first application of chromosome painting and fineSTRUCTURE algorithms and (2) presenting a quantitative analysis of recombination events of soft-core genes that revealed frequent recombination among housekeeping genes previously reported to be less prone to recombination.

It is important to keep in mind that sampling bias and sample size may limit our ability to interpret results related to population structure and recombination [20]. If this is the case for hpSWEurope phages, further studies are needed with increased sample size and more geographically diverse isolates.

Within a bacterial genome, prophages are considered to be part of the accessory genome and are not considered to be a part of the bacterial species’ core genome [40]. However, the phages themselves have their own pan-genome, although an accurate determination is challenging due to their high diversity. Our sample of *H. pylori* phages had a pan-genome composed of 75 genes, and a small soft-core genome comprising 13 % of the pan-genome genes, which is in agreement with the small-core genomes of mycobacteriophages [10], marine roseophages [41] and the phages that infect *Bacillus cereus* [42], *Escherichia coli* [43] and *Clostridium difficile* [44]. The percentage identity used for pan-genome determination is of paramount importance, and for *H. pylori* phages, we realized that it should not be higher than 70 % to avoid missing similar genes due to diversity. At one end of the pan-genome spectrum, there are 16 singleton genes in 11 genomes, which may have no selective benefit to the phage that carries them, or they could serve as a ‘gene nursery’, where novel genetic functions could develop [39], while at the other end, there are 10 soft-core genes, which should have essential roles in the phage life cycle. Indeed, these genes function in lysogeny (integrase); cell lysis (holin); DNA packaging (terminase, portal protein) and ejection (terminase); DNA replication, recombination and nucleotide excision repair; RNA transcription and splicing (helicase); and as structural components (structural protein). Furthermore, soft-core genome sequences are useful for clarifying the evolutionary relationships among prophages [45].

Analysis of recombination at the gene level revealed that the *H. pylori* phage soft-core genes were equally highly prone to recombination, indicating a continuous shuffle of sequences shaping phage genomes. Thus, the previous proposal that genes evolve at different speeds, with integrase evolving very quickly and holin nearly standing still [37], does not appear to be true for *H. pylori* phages [21]. A comparison to the analysis of the Earth’s virome suggested that *H. pylori* phages are among the most recombinogenic phages on Earth. The high recombination rate in *H. pylori* phages suggests the existence of frequent interactions between episomal phages and prophages, or the existence of recombination between multiple infected strains carrying prophages. Such frequent recombination may aid in the escape from bacterial immunity, especially given that restriction and modification systems are extremely abundant in *H. pylori* [46, 47]. Although 70 % of the *H. pylori* prophages were predicted to be active, it might also indicate that some of them are in decay due to frequent recombination, making the difference in the recombination intensity of genes undetectable. In other words, recombination may also protect the bacterial host by inactivating prophages, suggesting a new aspect of recombination that should be further examined in subsequent studies.

## Data bibliography

1. Vale FF. Sequence Read Archive (SRA), SRP064706 to SRP064710, SRP071062,SRP071067, SRP071271, SRP071274, SRP071276 to SRP071280, SRP071282, SRP071284, SRP071289 to SRP071296 and SRP072438 to SRP072441 (2016).

2. Vale FF. Genbank, KX119174 to KX119206 (2016).

## Supplementary Data

Supplementary File 1

## References

[1] Eppinger M, Baar C, Linz B, Raddatz G, Lanz C (2006). Who ate whom? adaptive *Helicobacter genomic* changes that accompanied a host jump from early humans to large felines. PLoS Genet.

[2] Uchiyama J, Takeuchi H, Kato S-ichiro, Takemura-Uchiyama I, Ujihara T (2012). Complete genome sequences of two *Helicobacter pylori* bacteriophages isolated from Japanese patients. J Virol.

[3] Luo C-H, Chiou P-Y, Yang C-Y, Lin N-T (2012). Genome, integration, and transduction of a novel temperate phage of *Helicobacter pylori*. J Virol.

[4] Vale FF, Nunes A, Oleastro M, Gomes JP, Sampaio DA (2017). Genomic structure and insertion sites of *Helicobacter pylori* prophages from various geographical origins. Sci Rep.

[5] Lehours P, Vale FF, Bjursell MK, Melefors O, Advani R (2011). Genome sequencing reveals a phage in *Helicobacter pylori*. mBio.

[6] Vale FF, Vadivelu J, Oleastro M, Breurec S, Engstrand L (2015). Dormant phages of *Helicobacter pylori* reveal distinct populations in Europe. Sci Rep.

[7] Thorell K, Yahara K, Berthenet E, Lawson DJ, Mikhail J (2017). Rapid evolution of distinct *Helicobacter pylori* subpopulations in the Americas. PLoS Genet.

[8] Paez-Espino D, Eloe-Fadrosh EA, Pavlopoulos GA, Thomas AD, Huntemann M (2016). Uncovering earth's virome. Nature.

[9] Paez-Espino D, Sharon I, Morovic W, Stahl B, Thomas BC (2015). CRISPR immunity drives rapid phage genome evolution in *Streptococcus thermophilus*. mBio.

[10] Pedulla ML, Ford ME, Houtz JM, Karthikeyan T, Wadsworth C (2003). Origins of highly mosaic mycobacteriophage genomes. Cell.

[11] Moodley Y, Linz B, Bond RP, Nieuwoudt M, Soodyall H (2012). Age of the association between *Helicobacter pylori* and man. PLoS Pathog.

[12] Thorell K, Lehours P, Vale FF (2017). Genomics of *Helicobacter pylori*. Helicobacter.

[13] Berthenet E, Sheppard S, Vale FF (2016). Recent "omics" advances in *Helicobacter pylori*. Helicobacter.

[14] Secka O, Vale FF, Buissonnière A, Thomas JE, Mégraud F (2017). Phylogeographic agreement between prophage and bacterial housekeeping genes in *Helicobacter pylori* strains from The Gambia. Helicobacter.

[15] Vale FF, Matos APA, Carvalho P, Vítor JMB (2008). *Helicobacter pylori* Phage Screening. Microsc Microanal.

[16] Vitoriano I, Vítor JMB, Oleastro M, Roxo-Rosa M, Vale FF (2013). Proteome variability among *Helicobacter pylori* isolates clustered according to genomic methylation. J Appl Microbiol.

[17] Silva B, Nunes A, Vale FF, Rocha R, Gomes JP (2017). The expression of *Helicobacter pylori tfs* plasticity zone cluster is regulated by pH and adherence, and its composition is associated with differential gastric IL-8 secretion. Helicobacter.

[18] Delahay RM, Croxall NJ, Stephens AD (2018). Phylogeographic diversity and mosaicism of the *Helicobacter pylori tfs* integrative and conjugative elements. Mob DNA.

[19] Kyrillos A, Arora G, Murray B, Rosenwald AG (2016). The presence of phage orthologous genes in *Helicobacter pylori* correlates with the presence of the virulence factors *CagA* and *VacA*. Helicobacter.

[20] Yahara K, Furuta Y, Oshima K, Yoshida M, Azuma T (2013). Chromosome painting *in silico* in a bacterial species reveals fine population structure. Mol Biol Evol.

[21] Meier-Kolthoff JP, Uchiyama J, Yahara H, Paez-Espino D, Yahara K (2018). Investigation of recombination-intense viral groups and their genes in the earth's virome. Sci Rep.

[22] Hu B, Xie G, Lo C-C, Starkenburg SR, Chain PSG (2011). Pathogen comparative genomics in the next-generation sequencing era: genome alignments, pangenomics and metagenomics. Brief Funct Genomics.

[23] Clokie MR, Millard AD, Letarov AV, Heaphy S (2011). Phages in nature. Bacteriophage.

[24] Pope WH, Bowman CA, Russell DA, Jacobs-Sera D, Asai DJ (2015). Whole genome comparison of a large collection of mycobacteriophages reveals a continuum of phage genetic diversity. Elife.

[25] Uchiyama J, Takeuchi H, Kato S-ichiro, Gamoh K, Takemura-Uchiyama I (2013). Characterization of *Helicobacter pylori* bacteriophage KHP30. Appl Environ Microbiol.

[26] Katoh K, Standley DM (2013). MAFFT multiple sequence alignment software version 7: improvements in performance and usability. Mol Biol Evol.

[27] Song W, Sun H-X, Zhang C, Cheng L, Peng Y (2019). Prophage Hunter: an integrative hunting tool for active prophages. Nucleic Acids Res.

[28] Lawson DJ, Hellenthal G, Myers S, Falush D (2012). Inference of population structure using dense haplotype data. PLoS Genet.

[29] Yahara K, Didelot X, Ansari MA, Sheppard SK, Falush D (2014). Efficient inference of recombination hot regions in bacterial genomes. Mol Biol Evol.

[30] Page AJ, Cummins CA, Hunt M, Wong VK, Reuter S (2015). Roary: rapid large-scale prokaryote pan genome analysis. Bioinformatics.

[31] Mills R, Rozanov M, Lomsadze A, Tatusova T, Borodovsky M (2003). Improving gene annotation of complete viral genomes. Nucleic Acids Res.

[32] Hudson RR, Kaplan NL (1985). Statistical properties of the number of recombination events in the history of a sample of DNA sequences. Genetics.

[33] Cai JJ (2008). PGEToolbox: a Matlab toolbox for population genetics and evolution. J Hered.

[34] Librado P, Rozas J (2009). DnaSP V5: a software for comprehensive analysis of DNA polymorphism data. Bioinformatics.

[35] Yahara K, Furuta Y, Morimoto S, Kikutake C, Komukai S (2016). Genome-wide survey of codons under diversifying selection in a highly recombining bacterial species, *Helicobacter pylori*. DNA Res.

[36] Goerke C, Pantucek R, Holtfreter S, Schulte B, Zink M (2009). Diversity of prophages in dominant *Staphylococcus aureus* clonal lineages. J Bacteriol.

[37] Ackermann HW, Elzanowski A, Fobo G, Stewart G (1995). Relationships of tailed phages: a survey of protein sequence identity. Arch Virol.

[38] Reuter S, Corander J, de Been M, Harris S, Cheng L (2015). Directional gene flow and ecological separation in *Yersinia enterocolitica*. Microb Genom.

[39] Hatfull GF, Hendrix RW (2011). Bacteriophages and their genomes. Curr Opin Virol.

[40] Ali A, Naz A, Soares SC, Bakhtiar M, Tiwari S (2015). Pan-genome analysis of human gastric pathogen *H. pylori*: comparative genomics and pathogenomics approaches to identify regions associated with pathogenicity and prediction of potential core therapeutic targets. Biomed Res Int.

[41] Chan JZ-M, Millard AD, Mann NH, Schäfer H (2014). Comparative genomics defines the core genome of the growing N4-like phage genus and identifies N4-like Roseophage specific genes. Front Microbiol.

[42] Geng P, Tian S, Yuan Z, Hu X (2017). Identification and genomic comparison of temperate bacteriophages derived from emetic *Bacillus cereus*. PLoS One.

[43] Petrov VM, Ratnayaka S, Nolan JM, Miller ES, Karam JD (2010). Genomes of the T4-related bacteriophages as windows on microbial genome evolution. Virol J.

[44] Ramírez-Vargas G, Goh S, Rodríguez C (2018). The novel phages phicd5763 and phicd2955 represent two groups of big plasmidial siphoviridae phages of *Clostridium difficile*. Front Microbiol.

[45] Uchiyama I, Albritton J, Fukuyo M, Kojima KK, Yahara K (2016). A novel approach to *Helicobacter pylori* pan-genome analysis for identification of genomic islands. PLoS One.

[46] Vale FF, Mégraud F, Vítor JMB (2009). Geographic distribution of methyltransferases of *Helicobacter pylori*: evidence of human host population isolation and migration. BMC Microbiol.

[47] Vale FF, Vítor JMB (2007). Genomic methylation: a tool for typing *Helicobacter pylori* isolates. Appl Environ Microbiol.

