# Supplementary material for: Analysis of genetic recombination and the pan-genome of a highly recombinogenic bacteriophage species
Source: Microb Genom. 2019 Jul 16;5(8):e000282. doi: 10.1099/mgen.0.000282 (PMC6755498; doi:10.1099/mgen.0.000282)
Supplement: Supplementary File 1 [file mgen-5-282-s001.pdf]

Analysis of genetic recombination and the pan-genome of a highly recombinogenic bacteriophage species

Supplementary Material

Parameters for pan-genome determination

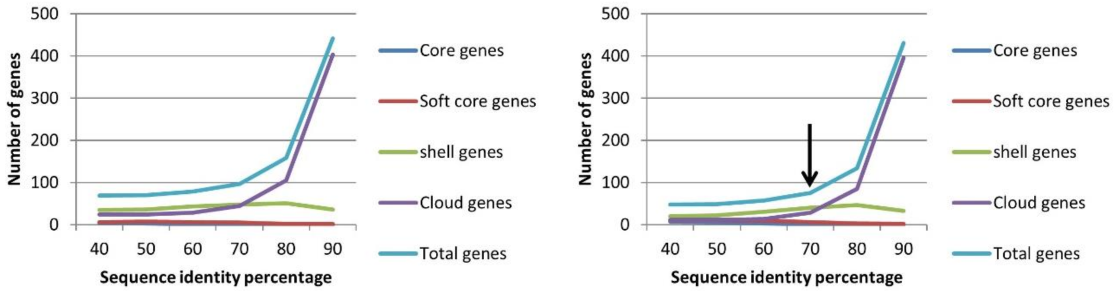

Figure S1. Summary of statistics output using Roary [1] for sequence identify percentage from 40 to 90 using intervals of 10. Left panel paralog enable and right panel paralog disable. The number of total genes exponentially increases for sequence identity percentages ≥ 70. The arrow points for the parameters used to select the soft-core genome of *H. pylori* prophages (sequence identity percentage = 70; paralog disable).

Visual examination of the genome alignment and a soft-core gene

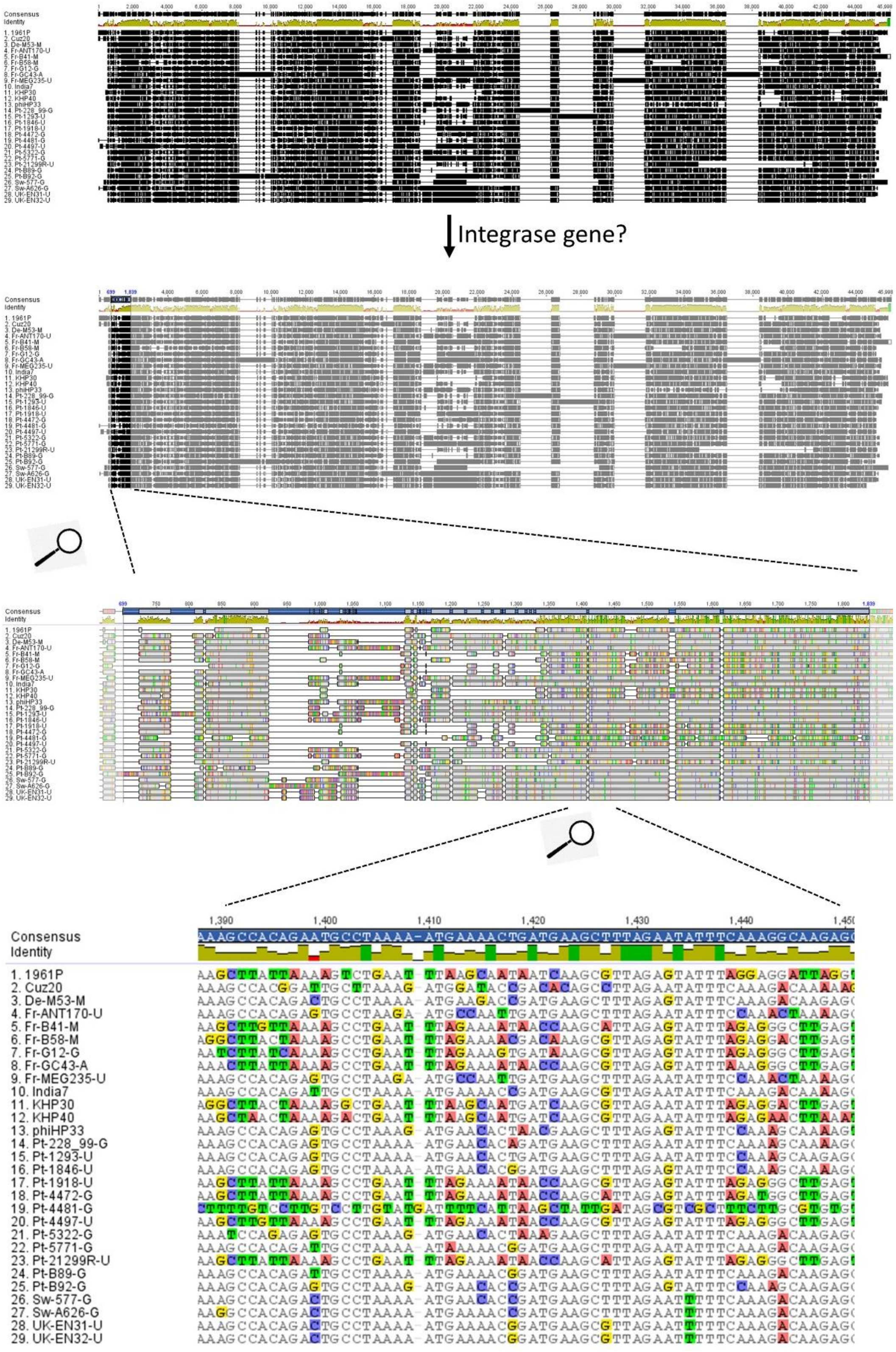

Figure S2. Successive close-ups identifying the position of the integrase gene in the phage multiple genome alignment (produced with MAFFT) in a synteny block. Top bar in the "Identity" track represents the color-coded consensus identity: green: 100% identity, greenish-brown: at

least 30% and under 100% identity, red: below 30% identity. The images were produced with Geneious 8.1.9. (a) Whole genome alignment. (b) Location of the integrase gene (black). (c) Zooming-in to the integrase gene. The black rectangles indicate regions with lower sequence similarity to the consensus than those illustrated by the light grey rectangles. (d) Zooming-in to the middle of the integrase gene.

Genetic flux among phage populations

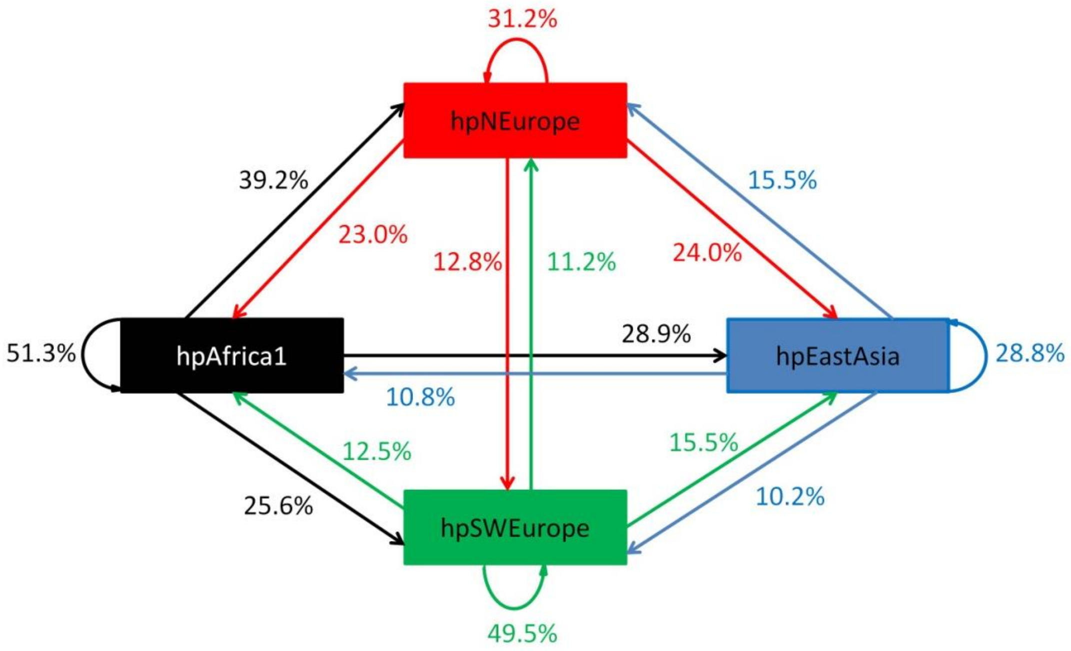

Figure S3. Genetic flux among phage populations. Exit arrows represents the average proportion of DNA chunks donated by a population to other population, i.e., entry arrows represent the average proportion of DNA chunks received from each population (for each genome the sum of all entry arrows is 100%; the percentages shown are averages for genomes from the same population and thus do not sum 100%, plus Hac phage is not considered).

Bacterial recombination

There is a clear evidence of recombination among *H. pylori* genomes, especially among genomes from the same population, i.e., hpEastAsia, hpEurope or hpAfrica1 (black squares in figure 3). Moreover, there is finer observable clustering, hpAfrica1 presents two subgroups, hpEurope presents 4 subgroups and, interestingly hpEurope isolates from North Europe (UK and Sweden) cluster with hpEastAsia genomes, forming an additional subgroup. In the case of bacterial genomes *H. acynonichis* clearly form an outgroup, receiving DNA chunks from all populations in an undistinguished manner.

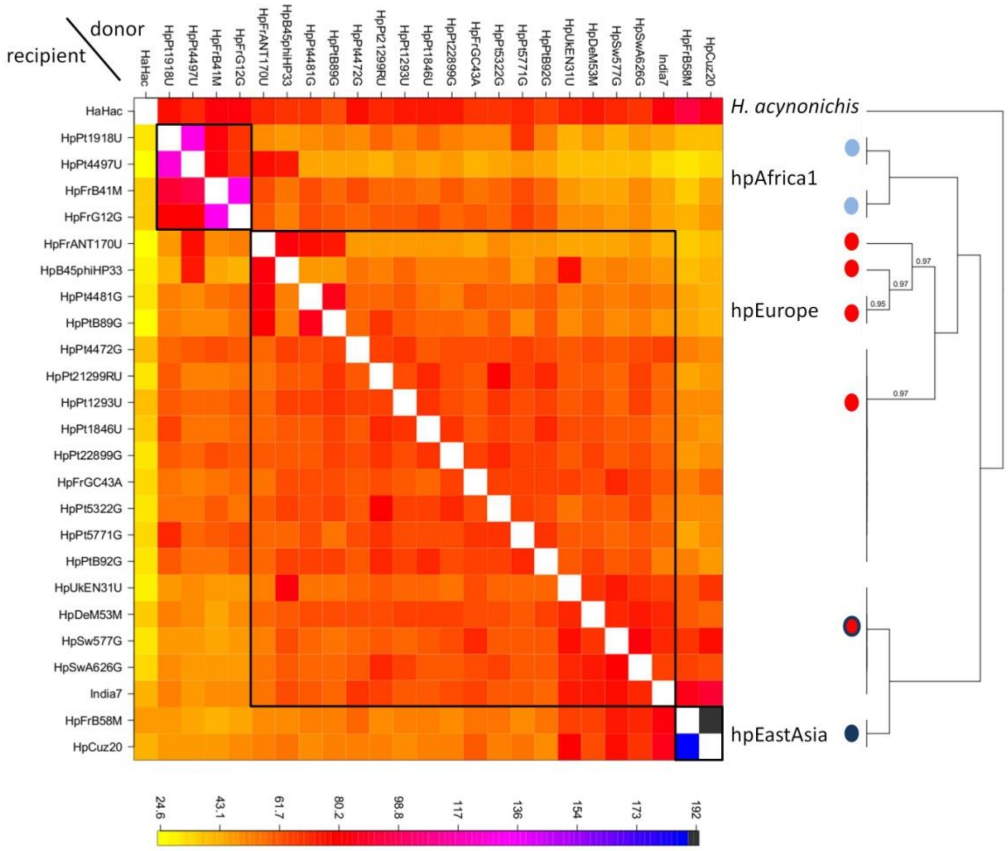

Figure S4. Co-ancestry matrix of *H. pylori* genomes that carry prophages. Each lane represents a *H. pylori* genome showed on the right. The color of each square of the matrix represents the expected number of chunks imported from a donor genome (column) to a recipient genome (row). These genomes are classified by MultiLocus Sequence Typing showed on the left together with a tree obtained from fineSTRUCTURE evidencing the subgroups of each population. The subgroups of each populations are evidenced by colored circles: hpEastAsia - dark blue, hpAfrica1 - light blue, hpEurope - red (hpEurope from northern Europe and India7, hpAsia2, clustered with hpEastAsia - red with blue delimiting circle).

Prediction of activity of *H. pylori* prophages

Table S1. Prophage Hunter [2] prediction of prophage activity.

Genome	Similarity matching not incorporated		Similarity matching incorporated	
	Category	Score	Category	Score
Hac	Ambigous	0,52	Ambigous	0,52
Fr-B58-M	Active	0,84	Ambigous	0,72
Pt-B92-G	Active	0,83	Ambigous	0,66
Pt-212-99R-U	No matching records		Active	0,93
Pt-1293-U	Active	0,9	Active	0,89
Pt-5771-G	Ambigous	0,69	Active	0,98
Pt-B89-G	No prophage found		Active	0,9
Pt-5322-G	No matching records		Ambigous	0,72
Fr-ANT170-U	No matching records		Active	0,83
Fr-MEG235-U	Ambigous	0,61	Ambigous	0,76
Pt-1846-U	Ambigous	0,59	Active	0,86
Pt-228_99-G	Ambigous	0,78	Ambigous	0,74
phiHP33	No matching records		Active	0,86
Pt-4481-G	Ambigous	0,58	Ambigous	0,67
UK-EN31-U	Ambigous	0,71	Ambigous	0,64
UK-EN32-U	Active	0,88	Ambigous	0,75
De-M53-M	Active	0,94	Active	0,92
India7	Active	0,9	Ambigous	0,67
Cuz20	Active	0,86	Ambigous	0,72
Sw-A626-G	Active	0,87	Active	0,82
Sw-577-G	Ambigous	0,69	Active	0,81
Fr-G12-G	Active	0,93	Active	0,81
Pt-4472-G	Ambigous	0,79	Ambigous	0,79
Fr-GC43-A	No matching records		Ambigous	0,63
Pt-1918-U	Active	0,98	Active	0,98
Pt-4497-U	Ambigous	0,76	Active	0,93
Fr-B41-M	Ambigous	0,58	Active	0,89

Prophage Hunter results run over bacterial genome carrying the prophages. A score > 0.8 indicates an active prophage, between 0.5–0.8 ambiguous, and <0.5 inactive. Note: Prophage Hunter operates over whole-genome sequences of bacteria (phages KHP30, KHP40 and 1691P are not included).

Reference *H. pylori* phage pangenome

Table S2. Clusters of orthologous genes.

	#	Annotation	Number of isolates carrying the gene	Reference pangenome	
				Phage	Protein ID
Soft-core genome	1	**Integrase**	29	1961P	AFC61901.1
	2	**Portal protein**	28	1961P	AFC61919.1
	3	**Structural protein**	28	1961P	AFC61916.1
	4	**Hypothetical protein/DNA associated**	28	1961P	AFC61904.1
	5	**DNA helicase**	28	1961P	AFC61907.1
	6	**Hypothetical protein #1**	28	1961P	AFC61927.1
	7	**Terminase**	28	1961P	AFC61920.1
	8	**Hypothetical protein #2**	27	Cuz20	ADO03377.1
	9	**Holin**	27	1961P	AFC61923.1
	10	**Hypothetical protein #3**	27	1961P	AFC61926.1
	11	Hypothetical protein	26	1961P	AFC61930.1
	12	Hypothetical protein	25	1961P	AFC61921.1
	13	Hypothetical protein	25	1961P	AFC61925.1
	14	Putative chromosome segregation protein	25	1961P	AFC61909.1
	15	Structure protein	24	1961P	AFC61918.1
	16	Structure protein	24	1961P	AFC61929.1
	17	Structure protein	23	1961P	AFC61915.1
	18	Hypothetical protein	23	DeM53M	ANT43224.1
	19	Hypothetical protein	23	1961P	AFC61925.1
	20	JHP1044-like mosaic region protein	22	1961P	AFC61910.1
	21	Hypothetical protein	22	Cuz20	ADO03395.1
	22	Structure protein	22	1961P	AFC61931.1
	23	Hypothetical protein	22	Cuz20	ADO03391.1
	24	Hypothetical protein	21	Cuz20	ADO03403.1
	25	Hypothetical protein	21	1961P	AFC61913.1
	26	DNA primase	20	Cuz20	ADO03398.1
	27	Hypothetical protein/DNA associated	20	1961P	AFC61904.1
	28	Hypothetical protein	17	Cuz20	ADO03375.1
	29	Putative tail fiber protein	12	FrB41M	ANT42641.1
	30	Hypothetical protein	11	1961P	AFC61906.1
	31	Terminase small subunit	11	1961P	AFC61932.1
	32	Tail fiber protein	11	FrB41M	ANT42640.1
	33	Putative tail assembly protein	11	FrB41M	ANT42642.1
	34	Hypothetical protein	10	DeM53M	ANT43203.1
	35	DNA primase	9	1961P	AFC61908.1
	36	Hypothetical protein	9	DeM53M	ANT43209.1
	37	Hypothetical protein	7	FrB41M	ANT42639.1
	38	Coiled-coil domain-containing protein	7	FrB41M	ANT42662.1

	39	Hypothetical protein	6	FrB41M	ANT42634.1
	40	Hypothetical protein	5	DeM53M	*
	41	Hypothetical protein	5	FrB41M	ANT42644.1
	42	Coiled-coil domain-containing protein	5	FrB41M	ANT42646.1
	43	Hypothetical protein	5	FrB41M	ANT42648.1
	44	Hypothetical protein	5	India7	ADU80393.1
	45	Hypothetical protein	5	phiHP33	AET85155.1
	46	Hypothetical protein	5	FrB41M	**
	47	Hypothetical protein	5	FrB41M	ANT42649.1
	48	Hypothetical protein	4	FrB41M	ANT42638.1
	49	Hypothetical protein	4	FrB41M	ANT42659.1
	50	Mobile element protein (tnpb)	4	FrGC43A	ANT42862.1
	51	IS605 transposase (tnpa)	4	FrGC43A	ANT42863.1
	52	Ishp608 transposase (orfb)	4	FrANT170U	ANT43079.1
	53	Mobile element protein (orfa)	4	FrANT170U	ANT43080.1
	54	Hypothetical protein	3	FrB41M	ANT42660.1
	55	Hypothetical protein	3	KHP40	***
	56	IS607 Mobile element protein (tnpb)	2	FrB58M	ANT42794.1
	57	Hypothetical protein	2	FrANT170U	ANT43066.1
	58	First ORF in transposon ISC1904 (tnpa)	2	FrB58M	ANT42793.1
	59	Hypothetical protein	2	UKEN31U	ANT42487.1
Singletons	60	Structure protein	1	1961P	AFC61917.1
	61	Hypothetical protein	1	1961P	AFC61922.1
	62	Putative Hac prophage II protein	1	Cuz20	ADO03406.1
	63	Hypothetical protein	1	Cuz20	ADO03385.1
	64	Nitogen-activated protein kinase 1	1	FrB41M	ANT42661.1
	65	Hypothetical protein	1	FrB58M	ANT42802.1
	66	Hypothetical protein	1	FrG12G	ANT42822.1
	67	Hypothetical protein	1	FrGC43A	ANT42857.1
	68	Hypothetical protein	1	FrGC43A	ANT42859.1
	69	Hypothetical protein	1	FrGC43A	ANT42886.1
	70	Putative Hac prophage II protein	1	India7	ADU80392.1
	71	Hypothetical protein	1	India7	ADU80415.1
	72	Hypothetical protein	1	KHP40	****
	73	Hypothetical protein	1	Pt1293U	ANT43103.1
	74	Hypothetical protein	1	Pt22899G	ANT42522.1
	75	Hypothetical protein	1	PtB92G	ANT42925.1

Newly detected by Prokka annotation:
*ATGATTAAAAAGCCTCACCTTGACCATACACAAGCCCTTTTGTCTTATGTGTCAGGGTCTCTTGGCACACTTAAAAAGTGTTTTTATGGGTTAGAAATATTCTCTAACCCATTGATTGAT

**ATGAGACAGCATGGAATAAATCCAAACGATCCTATAGAAGAACTCTTAAGAACTACAAGAAAGAAGCTGATGCTAAGAGAAAGCAGTTACAAAAAGCTAAAAAGGATGAAAAGAAAAAGGCGTTATACATTTGCAAGAGTAGTCCGTTTAGCTATTTTAGAGGGTATTAGGAAGTTTCAAAAAGAAAACCTATTTTTGAACTTGCAAATGCAAAATTTTGAGCTCGCAATAAGCATTTTAGAACAAAAAACAAAAAGAGCCAGAAATAAGGGAAAAAATGCAGAATTTGATAATGAATAG

***ATGGCGAAAATACCCAAGCGCGCAGAATTTGGATTATTGAAAATGAAGTTTTTAGAAATGTTAGCGATTAGCGTTTTGATTTTCATTTTAGGGATTAGCTTTATTTTAGCGGTTTATTTTTCTGTAGAGGCGTTATGCTATGGATAA

****ATGCGGGTTTTGTTAACCCATTTTTGGGATTTGTTGGGTGTTTTTGTGTTTGTTATTAGATTATTTCTATATATTCCAAAAAACAAAAACAATAACCCCGATAAAGAGCTAAATCAAAAAAAAGCGGTTACTATAAAAGCCCTACAATAG

References

1. Page AJ, Cummins CA, Hunt M, Wong VK, Reuter S, Holden MT, Fookes M, Falush D, Keane JA, Parkhill J. Roary: rapid large-scale prokaryote pan genome analysis. Bioinformatics 2015;31:3691-3693.

2. Song W, Sun HX, Zhang C, Cheng L, Peng Y, Deng Z, Wang D, Wang Y, Hu M, Liu W, Yang H, Shen Y, Li J, You L, Xiao M. Prophage Hunter: an integrative hunting tool for active prophages. Nucleic Acids Res 2019; pii: gkz380.
